# Isolated colon ischemia with norovirus infection in preterm babies: a case series

**DOI:** 10.1186/1752-1947-7-108

**Published:** 2013-04-17

**Authors:** Gloria Pelizzo, Ghassan Nakib, Ilaria Goruppi, Mario Fusillo, Federico Scorletti, Simonetta Mencherini, Gian Battista Parigi, Mauro Stronati, Valeria Calcaterra

**Affiliations:** 1 Department of Mother and Child Health, Pediatric Surgery Unit, IRCCS Policlinico San Matteo Foundation and University of Pavia, Pavia, 27100, Italy; 2 Department of Anaesthesiology and Intensive Care, IRCCS Policlinico San Matteo Foundation and University of Pavia, Pavia, 27100, Italy; 3 Department of Mother and Child Health, Neonatal Intensive Care Unit, IRCCS Policlinico San Matteo Foundation, Pavia, 27100, Italy; 4 Department of Internal Medicine, University of Pavia and Department of Mother and Child Health, Pediatric Unit, IRCCS Policlinico San Matteo Foundation, Pavia, 27100, Italy

**Keywords:** Colonic perforation, Colonic stenosis, Norovirus infection, Premature

## Abstract

**Introduction:**

Norovirus infection with necrotizing enterocolitis has so far been reported as a specific tropism of the small bowel in premature newborns.

**Case presentation:**

Three cases of premature newborns presenting with extensive isolated colonic ischemia due to norovirus infection are reported.

Patient 1 was a Caucasian girl with a gestational age of 29+2 weeks. She had sudden onset of abdominal distension on the 30th day of life. Radiological signs of colonic pneumatosis were present 48 hours before perforation and stool analysis was positive for norovirus. On the 34th day, free air was detected on plain abdominal X-ray. At laparotomy, stenosis, necrosis and perforations involved the whole colon. The patient underwent ileostomy. A large colon resection and ileosigmoid anastomosis were done 3 months later.

Patient 2 was a Caucasian boy with a gestational age of 28+3 weeks. On the 19th day, bloody stools with abdominal distension appeared. Stool analysis resulted positive for norovirus. A plain abdominal X‐ray showed distended bowel loops. Antibiotic treatment was started. On the 32nd day due to the progressive deterioration of clinical conditions and the appearance of colic pneumatosis, a laparotomy was performed. Severe damage of the transverse colon and multiple areas of necrosis were found. Terminal ileostomy was performed. Six months later surgery consisted of mid-transverse colon resection as far as the splenic flexure, colocolic anastomosis and closure of ileostomy.

Patient 3 was a Caucasian boy with a gestational age of 30 weeks. On the 44th day bloody-mucous stools appeared and stool analysis was positive for norovirus infection. Even with institution of antibiotic therapy clinical abdominal radiologic signs of colonic pneumatosis of the upper right quadrant were found. At the 70th day an explorative laparotomy showed dilated bowel loops and stenotic right colon and ileostomy was mandatory. Partial colectomy was later necessary and ileocolic anastomosis was performed.

**Conclusion:**

We hypothesize that norovirus infection may be responsible for severe, distinctive colonic lesions, even in premature newborn infants.

## Introduction

Norovirus (NoV) gastroenteritis is generally mild and of short duration in healthy infants [1], but it has been reported as being fatal in vulnerable populations, such as newborns, especially when it occurs in association with necrotizing enterocolitis (NEC) [2,3]. Although NEC does not appear to be caused by one specific pathological agent, NoV is assumed to be responsible in clustered outbreaks with similar clinical courses [3]. Patterns of pathology reported for NoV in association with NEC in neonatal intensive care units (NICUs) have normally involved the small intestine; so far there are no reports of exclusive compromise of the colon.

We present three cases of NoV infection with massive ischemia confined to the colon.

## Case presentation

The three babies, described in Table 1, were observed within a 3-month period.

**Table 1:** Patients’ characteristics

**#**	**Sex**	**GA weeks**	**Ethnicity**	**Birth weight**	**Apgar 1**^**′**^**‐5**^′^	**CPAP support at birth**	**Day onset symptoms**	**Day 1st surgery**	**Type of surgery**	**Follow-up months**
**1**	F	29+2	Caucasian	1145g	8‐9	Yes	20	34	1st ileostomy	A&W
									2nd total colectomy	12
**2**	M	28+3	Caucasian	1180g	6‐8	Yes	19	34	1st ileostomy	A&W
									2nd partial	
									colectomy	11
**3**	M	30+0	Caucasian	1134g	1‐9	Yes	44	71	1st ileostomy	A&W
									2nd partial colectomy +	11
									direct anastomosis	

A&W = alive and well; CPAP = continuous positive airway pressure; GA = gestational age.

### Case 1

**Figure 1:**
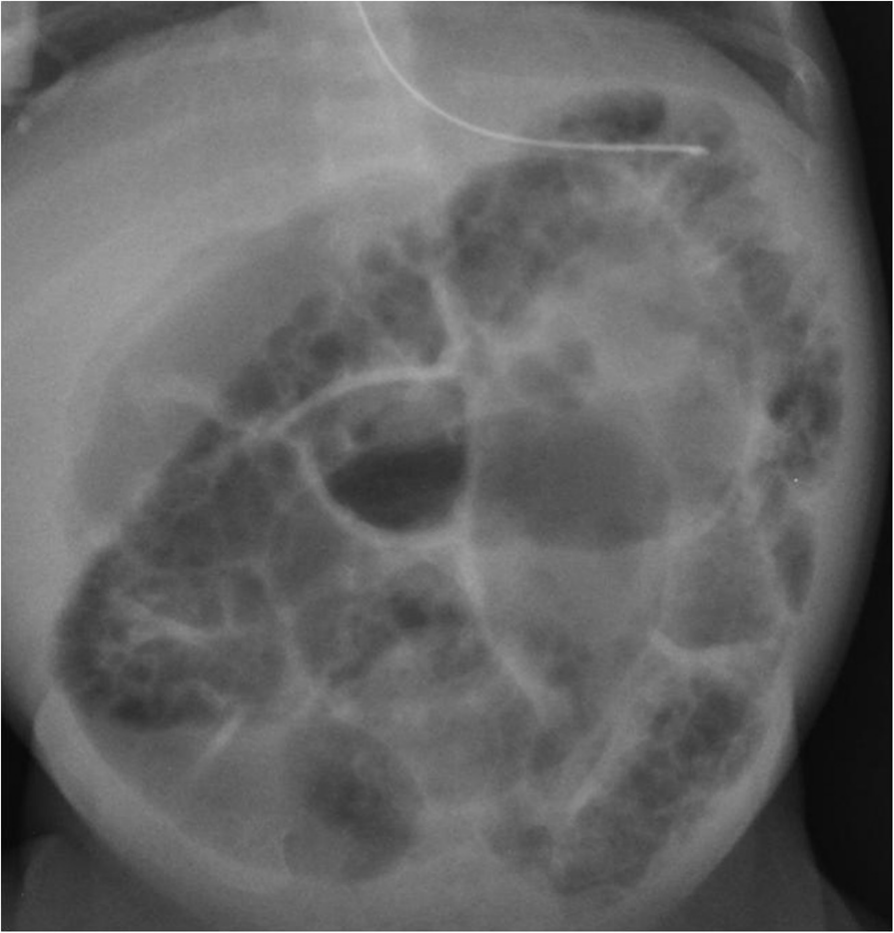
Patient 1 – Pneumatosis involving the whole colon.

Patient 1 was a Caucasian girl, gestational age 29+2 weeks with a birth weight of 1145 g. The newborn was maintained under ventilatory support for 10 days. Sudden onset of abdominal distension appeared on the 30th day of life. A plain abdominal X‐ray showed distension of ileal loops. Although there was no free air, pneumatosis involving the whole colonic length was noted (Figure 1). Antibiotic therapy was supplemented and total parenteral nutrition started. On the 33rd day, her clinical conditions worsened with the appearance of greyish skin, bilious gastric residuals and bloody‐mucous stools. Air‐fluid levels were observed on abdominal X-ray. Stool samples, collected 48 to 72 hours after onset of symptoms and stored at +4°C, were assayed by enzyme-linked immunosorbent assay (ELISA) and real-time polymerase chain reaction (RT-PCR) technique. The only pathological agent detected was NoV.

On the 34th day, free air was detected in her abdomen. At laparotomy, the whole jejunum and ileum were of normal appearance, whereas her colon demonstrated segmental stenosis, several areas of necrosis and perforations. Total colectomy plus ileostomy was carried out. Ileosigmoid anastomosis was performed 10 weeks later. On follow up the baby was free from early chronic diarrhea. Only one episode of dehydration due to intestinal infection was reported.

### Case 2

Patient 2 was a Caucasian boy, gestational age 28+3 weeks, with a birth weight of 1180g. Prolonged continuous positive airway pressure for respiratory distress at birth was maintained. On the 19th day, bloody stools and abdominal distension appeared along with a general deterioration in conditions. Fasting and antibiotics were implemented because of a mild elevation in inflammatory lab markers: white blood cells (WBC) 13.000/mm^3^ and C-reactive protein (CRP) 4.3mg/dL. Stool analysis (see Patient 1) was positive for NoV. A plain abdominal X‐ray showed distended bowel loops with no free air, but was suspicious for initial colic pneumatosis. On the 32nd day enteral nutrition was restarted, but was followed by significant abdominal distension. An exploratory laparotomy carried out for progressive deterioration showed a normal but distended small intestine. His transverse colon was severely inflamed and thickened and congested and stenotic areas were found. Terminal ileostomy was mandatory to protect the colon.

**Figure 2:**
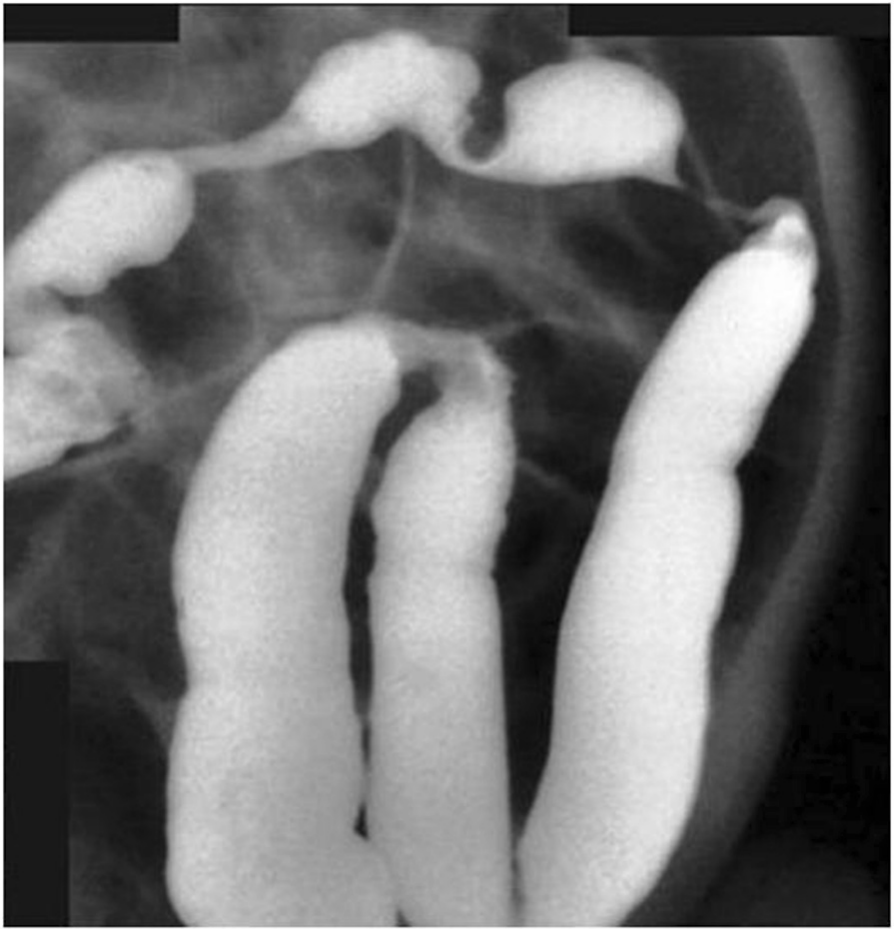
Patient 2 – Barium enema: tight stenosis at the splenic flexure, with multiple strictures of the transverse colon.

Six months later it was possible to close the stoma, but the mid‐transverse colon had to be resected as far as the splenic flexure due to multiple stenoses visible at barium enema (Figure 2). Histology showed fibrosis of the large bowel wall without evidence of necrosis. Hyperplastic vessels with thickened walls were observed both in the submucosa and in the subserosa layers.

### Case 3

Patient 3 was a Caucasian boy, gestational age 30+0 weeks, with a birth weight of 1134 g. On the 18th day he was intubated for three days due to suspected sepsis. On the 44th day bloody‐mucous stools appeared and the stool analysis was positive for NoV infection (ELISA, RT-PCR analysis). Fasting and antibiotic treatment were implemented for two weeks. At the end of this period abdominal distension and bloody-mucous stools suddenly reappeared. On abdominal X‐ray diffuse bowel loops distension and upper right quadrant colonic pneumatosis were noted but no free air. WBC and PCR were slightly elevated at 14.000/mm^3^ and 6.2mg/dL respectively. At the 70th day explorative laparotomy showed dilated but normal small bowel loops. The right colon was stenotic. Ileostomy was performed. Four weeks later, partial colectomy was necessary before performing a direct ileocolic anastomosis. Macroscopically the colon showed thickening and fibrosis of the bowel wall without evidence of necrosis. At histology, hyperplastic vessels with thickened walls were observed both in the submucosa and in the subsierosa.

## Discussion

NoV, which belongs to the *Caliciviridae* family, is a highly contagious virus and is thought to be one of the most important causes of nonbacterial acute gastroenteritis in all ages in developing as well as in developed countries [2]. NoV associated with gastroenteritis in infants usually presents with mild, self-limiting clinical signs like diarrhea with stools that are loose or watery but without mucus; and slightly elevated inflammatory lab tests [1]. However, it is also found in up to 40% of preterm babies with NEC infections [4], although the risk of patients with NoV developing NEC remains unknown.

So far, NEC in association with the NoV tropism has seemed to affect only the upper intestinal tract [5], with histological findings of villous atrophy, crypt cell hyperplasia and cytoplasmic vacuolization confined to the jejunum and ileum. No previous reports of enteric NoV infection have included descriptions of the histological characteristics of the colonic wall; and no studies have been performed to evaluate the possibility of colon involvement. As far as we know there are no reports in the literature on NEC involving the colon in cases of NoV infection in humans.

By contrast, in all three of our cases, intestinal insult was limited to the colon; a localization that resulted in extensive damage necessitating long colonic resections. Selective colonic tropism has recently been described in animal models as being associated with a particular murine NoV genotype [6]; with glycan binding during NoV colonic infection thought to play a key role [7]. The dynamic change in mucosal glycosaminoglycan composition described for premature babies may be involved.

NoV infection in premature infants shows a striking variety of clinical courses [5,8]. There is a serious risk of underestimating the gravity of the condition when only the colon is involved, because the clinical course is apparently less acute and severe than for small intestine involvement. In our cases, the presurgical course was characterized by long, alternating periods of clinical improvement and deterioration unexpectedly culminating in colonic perforation. This history was most probably due to the long duration of the subacute progression of ischemic damage confined to the colon. It is possible that colonic vascularization is maintained for a longer time than small bowel vascular support. When the colonic vascular supply between the right and left systems is finally totally compromised, several stenoses might appear more or less simultaneously along the whole colon in newborns. The histology of the spontaneous focal colonic perforations found at surgery was characterized by the absence of inflammatory lesions, no apparent neutrophilic infiltration, and no regenerative or ischemic changes like those found in necrotic tissues [9]. By the time pneumoperitoneum appeared, bowel necrosis and wide vascular lesions were already established and aggressive surgical measures were necessary.

Unfortunately there are no clear radiological signs for the monitoring of colonic involvement except for the appearance of colonic pneumatosis. The appearance of this sign justifies immediate surgical intervention, and the creation of an ileostomy as early as possible.

Three cases are obviously not enough to be able to postulate a new clinical entity caused by NoV infection in premature babies. Nevertheless, given their severity, we think it is worthwhile to draw surgeons’ attention to the following:

Bowel distension associated with NoV infection and clinical deterioration in preterm babies constitutes an indication for surgical surveillance and early surgical treatment;

Early treatment is mandatory. An ileostomy should probably be performed as soon as colonic pneumatosis is detected by X-ray;

Ileostomy is necessary to conserve colon integrity.

## Conclusions

To repeat our warning, NoV infection may have a tropism affecting the colonic district. In the literature the most commonly reported lesions with an exclusively colonic localization are spontaneous colonic perforation [10] whereas, as far as we know, there are no reports of injury solely to the colon associated with NoV infections. Further physiological investigation of the role and tropism of different viral and bacterial infective agents towards the colonic cells in premature babies may clarify when and how this apparently new clinical condition occurs.

Our case report raises many questions: is this particular condition related to the presence of NoV in the NICU? Is simultaneously occurring NEC a predisposing condition for developing colon involvement where there is NoV infection in the NICU?

In terms of how to deal with this elusive condition, rapid diagnosis is essential for favorable prognosis; total or partial colon resection cannot be avoided if the clinical status of the newborn is underestimated. However, prompt diagnosis is intrinsically difficult, so we recommend maintaining a low threshold for suspicion of NEC of the colon in premature babies, even in the presence of apparently mild symptoms associated with NoV.

## Consent

Written informed consent was obtained from the patients’ legal guardians for publication of this manuscript and any accompanying images. Copies of the written consents are available for review by the Editor-in-Chief of this journal.

## Competing interests

The authors declare that they have no competing interests.

## Authors’ contributions

GP performed the surgical support and the writing of the final manuscript. GN performed the surgical support and the draft preparation of the manuscript. IG performed the surgical support. MF performed the surgical support. FS performed the surgical support. SM performed the anesthesiological support. GBP performed the surgical support. MS performed the neonatal care. VC performed the draft preparation of the manuscript and the literature search. All authors read and approved the final manuscript.
